# Challenges and Experiences in Multicenter Prehospital Stroke Research: Narrative Data from the Rapid Intervention with Glyceryl Trinitrate in Hypertensive Stroke Trial-2 (RIGHT-2)

**DOI:** 10.1080/10903127.2023.2287171

**Published:** 2023-11-29

**Authors:** Mark Dixon, Julia Williams, Philip M. Bath, for the RIGHT-2 Investigators

**Affiliations:** a Stroke Trials Unit, Mental Health & Clinical Neuroscience, University of Nottingham, Queens Medical Centre, Nottingham, UK; b East Midlands Ambulance Service NHS Trust, Nottingham, UK; c Department of Paramedic Science, School of Health and Social Work, University of Hertfordshire, Hatfield, UK; d Stroke, Acute Medicine, Nottingham University Hospitals NHS Trust, Nottingham, UK

## Abstract

**Background:**

Ambulance services are increasingly research active and the Rapid Intervention with Glyceryl trinitrate in Hypertensive stroke Trial-2 (RIGHT-2) is the largest United Kingdom (UK) ambulance-based randomized controlled trial in stroke. We explore the complexities and challenges encountered during RIGHT-2.

**Methods:**

Five hundred and sixteen of 1487 paramedics from eight UK ambulance services serving 54 comprehensive or primary stroke care centers screened and consented 1149 patients presenting within 4 h of FAST-positive stroke and with systolic blood pressure >120 mmHg; participants were randomized to treatment with transdermal glyceryl trinitrate versus sham patch in the ambulance.

**Key findings:**

Working with multiple ambulance services demanded flexibility in the trial protocol to overcome variation in operating procedures to ensure deliverability. Many paramedics are novice researchers, and research concepts and practices are emerging including consent strategies in emergency stroke care. Regional variation in hospital participation and hours/days of operation presented paramedics with additional considerations prior to patient recruitment. The working hours of hospital research staff often do not reflect the 24/7 nature of ambulance work, which challenged deliverability until trial processes became fully embedded. Management of investigational medicinal product between ambulance stations, in-transit when on ambulance vehicles and on handover at hospital, necessitated an in-depth review to maintain accountability.

**Conclusion:**

RIGHT-2 demonstrated that although there are significant practical challenges to conducting multicenter ambulance-based research in a time-dependent environment, careful planning and management facilitated delivery. Lessons learned here will help inform the design and conduct of future ambulance-based trials.

## Introduction

Research activity in ambulance services is rapidly evolving. Most completed prehospital research trials have centered on life-threatening scenarios including cardiac arrest (1, 2), airway management (3, 4), stroke (5–8), and myocardial infarction (9, 10). Ambulance services are experiencing a shift in the nature and scope of the workload away from traditional emergency presentations toward social, urgent, and primary care; similarly, research is developing to support these frequent interactions, for example community-based referral for older adults who fall (11, 12).

However, few prehospital studies are randomized controlled trials and the challenges of implementation, conduct, and delivery of large, multicenter, and complex studies are only beginning to emerge (13–15). Reviews of obstacles to prehospital research concluded that ambulance services in the United Kingdom cover large geographical areas, often have small research departments, are increasingly interested in research and being research active, but have a workforce who lack experience of running or participating in complex trials (16, 17).

The nature of the prehospital environment gives rise to unpredictable and sometimes uncontrollable situations; further, there are ethical considerations specific to ambulance-based settings, such as obtaining informed consent in patients with time-critical presentations, additional data collection, and pressure placed upon paramedics to complete research-training activity lead to further challenges (18).

The registrant body for United Kingdom (UK) paramedics, Health and Care Professions Council, has raised the educational threshold from a vocational in-service certificate to university-based honors degree from September 2021 (19) inculcating exposure, insight, and understanding of research, methodologies, and evidence-based practice. Furthermore, research paramedic roles are emerging to support, deliver, and promote research within ambulance services, but many roles rely on externally funded trials to support short-term or temporary posts (16, 17).

In light of these factors, many ambulance studies have been small scale, in single sites, usually involving a sub-population of patients and clinicians that limits generalizability to wider clinical practice.

This narrative review examines ambulance-based research drawing upon experiences from the Rapid Intervention with Glyceryl trinitrate in Hypertensive stroke Trial-2 (RIGHT-2) (20). RIGHT-2 is the largest UK based ambulance-based trial in stroke. Eight of 13 UK ambulance services and 54 stroke centers participated over 32 months of recruitment (September 2015-October 2018) with 1,487 trained paramedics recruiting 1,149 patients.

## Methods

RIGHT-2 was a multicenter prospective, single-blind, parallel group randomized trial. It was developed as a phase III follow-on trial from the small-scale RIGHT pilot (7) and completed hospital trials that suggested early administration of glyceryl trinitrate (GTN) might improve functional outcome (21–23). Together with PIL-FAST (5), RIGHT suggested that trials in UK ambulance-based prehospital stroke were feasible but required testing across multiple sites. The protocol (24), statistical analysis plan (25), baseline characteristics of enrollees (26), main results (20), and some secondary findings (27–30) have been published previously. The eligibility criteria are described elsewhere (20, 24) but included adult patients with suspected stroke; a face, arm, speech, time (FAST) score of 2 or 3; systolic blood pressure of >120 mmHg; treatment commencement within 4 h of symptom onset; presentation to a trial-trained paramedic; and transfer to a participating hospital. Standard care procedures would otherwise apply.

RIGHT-2 was was funded by the British Heart Foundation (grant number CS/14/4/30972), approved by the UK regulator (Medicines and Healthcare Products Regulatory Agency, reference: 03057/0064/001–0001; Eudract 2015–000115–40), and national research ethics committee (IRAS: 167115), and was adopted by the National Institute for Health Research Clinical Research Network. Participants gave informed consent to participate in the study before taking part.

The investigational medical product (IMP) was manufactured by The Nottingham University Hospitals NHS Trust clinical trials pharmacy. Packs contained opaque sealed sachets with either a GTN or sham patch, and a gauze dressing for placing over the patch to provide additional participant blinding. Four sachets were contained within a larger plastic box (treatment pack) containing a patient information sheet, consent form, and ambulance case report form (CRF). Treatment packs were individually numbered and sealed with shrink-wrapped plastic for dispatch to ambulance service trust pharmacies for onward distribution to participating ambulance stations.

Patients were randomized to receive transdermal GTN 5 mg or a sham patch first in the ambulance and then daily for up to 3 days in hospital. For each eligible patient randomized in the trial, the paramedic who enrolled the patient completed a CRF that was additional to the trial consent form and the standard ambulance patient care record. This CRF captured information at baseline, during, and immediately after treatment, including demographics (age, sex), stroke (onset date/time), physiology (blood pressure, heart rate, oxygen saturation, glucose), and timings (randomization, patch application).

Hospitals continued care and provided data on assessment, diagnosis, intervention, recovery, and outcome, which was stored in a secure database accessible to researchers. Final follow-up at day 90 was performed centrally *via* telephone by a blinded assessor.

RIGHT-2 researchers collaborated with multiple National Health Service (NHS) stakeholders and organizations. The NHS is organized into Trusts, which are individually managed organizations each serving a specific geographical area or providing specialized areas of care. Hyperacute stroke facilities will be designated destinations within certain acute hospital trusts for patients assessed by ambulance staff as suffering suspected stroke. This may mean ambulance crews bypass a local emergency department without a stroke service in view of reaching definitive care.

Ambulance service provision in the UK is divided into 13 ambulance trusts, of which ten are regional services in England. Country-wide services operate across Wales, Scotland, and Northern Ireland. Separate arrangements are in place for islands and overseas territories (Isle of Wight, Isle of Man, Jersey, Guernsey, and Gibraltar). All ambulance clinicians follow the expert consensus clinical guidelines formed by the Joint Royal Colleges Ambulance Liaison Committee. Each service is responsible for its individual processes and procedures giving rise to variation between service provision and operating models.

As narrative enquiry aims to explore and conceptualize experiences (31), key challenges experienced are discussed offering insight, solutions, and recommendations based on lessons learned through the RIGHT-2 trial.

## Key Findings

Key findings of this narrative review are presented by theme based on the experience of the RIGHT-2 researchers from conception to publication. Learning identified across the following topics will be discussed: designing the trial, set-up, recruitment and training of paramedics in trial procedures, interactions with recruiting centers, management of IMP, and data collection (Table 1).

**Table 1.** Key lessons learned from the RIGHT-2 trial.

Very early engagement with ambulance services, clinical research networks, and hospital partners will support identification and mitigation for organizational nuances. Trial protocol to be developed with flexibility to respect ambulance service organizational differences yet maintaining impact and governance to deliver research objectives and outcomes. Multimodal trial training within ambulance services will encourage participation and engagement, respecting learning styles and offering opportunity to participate across large geographic areas across the 24/7 period. Facilitate technology use to support data collection and avoid duplication of tasks. Use QR codes, GPS, and web-based logs to support accountability of IMP.

### Designing the Trial

Originally, RIGHT-2 was designed to involve five UK NHS ambulance services (four in England, one in Scotland based on expressions of interest) and 30 hospitals with recruitment of 850 patients over 2 years.

Ambulance service internal organizational and operational factors often defined the specific localities targeted to participate, but also relied upon hospital stroke units within those regions to participate. We experienced double jeopardy where an ambulance service was interested but with no willing hospitals, and vice versa. Thus, we recognized and acknowledged that smaller pockets of participating locations would result in lower recruitment and a prolonged recruitment phase. Part way through the recruitment phase of the trial, approval was sought to increase participating sites to eight ambulance services and 54 hospitals across a total recruitment period of 32 months.

There were distinct strengths and challenges in both regional and small-locality participation. Only one ambulance trust was able to roll-out the trial across its whole geographic area as all ten acute stroke facilities in the region had capacity to participate. This offered wider participation and removed the additional step for paramedics to identify whether the nearest stroke center to the emergency scene was taking part in the trial prior to commencing randomization.

Conversely, small locality focus offered several advantages, such as to build momentum and engagement with the trial across a discrete number of ambulance stations. However, we recognized that this disadvantaged many paramedics from the opportunity to take part. Smaller localities allowed for greater oversight and control of trial processes including IMP management and data collation, especially relevant in view of the relative research-inexperience of participating paramedics at the time of the trial.

Participating locations were determined by local research teams within their respective ambulance trusts based on capacity, experience, geographic considerations of the stations, numbers of paramedics, and the location of participating hospitals. Similar considerations have been reported elsewhere (16).

During the set-up phase, one ambulance service who had previously expressed interest in participating subsequently felt that its capacity to deliver the protocol was impeded by poor stroke care compliance; this was despite considerable interest from two hospitals in the region. As a result, that region did not participate.

The trial management team experienced reluctance when negotiating with some hospital organizations, which was attributed to concurrent recruitment to other hyperacute or commercial stroke trials, and the potential loss of recruitment credits as co-enrollment was not possible. This forced smaller clusters of participation within ambulance service regions.

One hospital stopped (switched-off) recruitment to paramedics once they reached the contracted number of recruited patients, resuming routine practice. Unfortunately, several patients were enrolled and conveyed to this hospital after the close-down since paramedics knew about prior participation and in spite of them being briefed on the hospital’s withdrawal.

Further to this, a small number of hospitals also ‘switched-off’ recruitment to paramedics due, in part, to reaching recruitment accrual point targets (points awarded per patient recruited). Accruals were split equally between ambulance and hospital trusts; hence, paramedics could not enroll patients in the vicinity of these hospitals once targets had been reached. Since the trial ended, the importance of accrual points has been downplayed by the NIHR Clinical Research Network.

Switching “on and off” the ability for paramedics to recruit and convey to specific hospitals was viewed by paramedics to add significant complexity to time-critical decision-making at the scene and so reduced motivation to take part.

### Recruitment of Paramedics

Each ambulance service had a study-specific research paramedic, who coordinated the trial in that region. Paramedics from nominated ambulance stations were invited to participate voluntarily. Altogether, 1,487 paramedics were trained from 184 ambulance stations from eight ambulance services across England and Wales; of these, 516 paramedics recruited one or more patients (20).

Only one ambulance trust offered participation to paramedics across all 96 ambulance stations as all 10 stroke centers in the region took part. This maximized paramedic participation and widened opportunity for participant enrollment, fostering inclusivity across the region, but had to be balanced against cost-effectiveness of IMP production and balancing the provision of trial resources across 96 ambulance stations spanning six English counties.

Paramedic training in trial procedures commenced prior to trial launch in each ambulance service region to improve chances of early randomization. Training was delivered through a web-based online platform accessible to paramedics *via* computer, tablet, and smart-phone devices. Online training was favored due to the ease of access for paramedics who are frequently away from base ambulance stations during routine shift work.

At the time of trial launch, ambulance service information technology services did not allow access to the university-hosted training video on station-based computers due to firewall security restrictions. Information technology infrastructure was reported as a barrier, with workstations not having access to live presentations. Where this could not be overcome, some paramedics were required to complete this in their own time; this was met with mixed reviews, some recognizing the need for professional development and willingness to participate in research with others not wishing training to encroach on their personal time. More recently, studies have offered to support training with financial incentives *via* overtime payment or vouchers as it is difficult to abstract paramedics away from frontline response.

**Figure 1.**
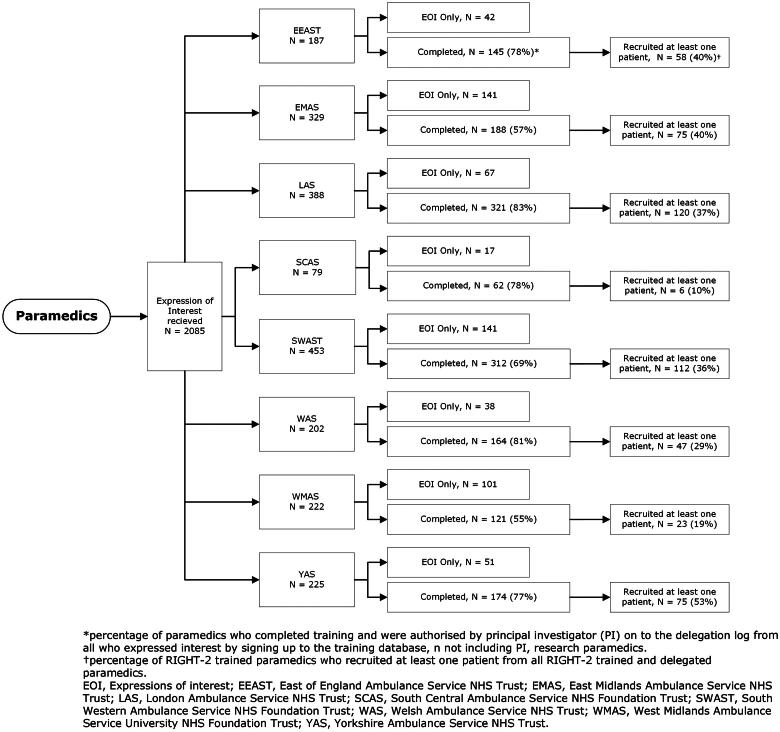
Paramedics participating in RIGHT-2.

The initial length of the training video (60 min) was highlighted as a limitation in precluding some paramedics from completing the training in one sitting, as breaks within operational shifts varied between 30 and 45 min. Many started the training but did not return to complete it (Figure 1). Following the video, understanding was confirmed with a knowledge check consisting of ten multiple choice questions. This was required to be completed and linked to the trial database where research paramedics authorized paramedics on to the trial delegation log. Working closely with research paramedics, due to the initial training length proving a barrier to participation, the training was re-worked and included bespoke service-specific procedures for local orientation. This included a mix of online and face-to-face sessions thereby widening participation and encouraging wider involvement.

At the time of the trial, no formal research training for paramedics existed. It was challenging for paramedics to access traditional good clinical practice (GCP) research training due to demands upon services and limited relevance of the content to the prehospital setting (32). The sponsor and UK’s competent authority (MHRA) agreed that a cut-down version of GCP would be acceptable, this including consent and IMP management. However, research exposure is now embedded throughout undergraduate paramedic education. Formal GCP training has been developed alongside research being recognized as a pillar of the College of Paramedics career framework (33).

### Trial Recruitment Hours

At trial launch, participant recruitment was limited to within Monday to Friday working hours to ensure that stroke researchers were available to meet participants and safeguard the transition of research materials between ambulance, emergency departments, and stroke units. This narrowed the recruitment window to approximately 30% of the week but enabled robust processes to be in place for the safe transfer of IMP, consent forms, and data sheets in often chaotic emergency environments.

Recognizing busy departments and high turnover of emergency department staff, local procedures were then embedded once the trial was fully understood in readiness to extend recruitment across the 24 h period. This removed the time restriction consideration from ambulance services reflecting real-time hyperacute stroke care and recruitment into hyperacute stroke research. Hospital sites were able to liaise with ambulance services to review recruitment hours at their discretion.

Some sites opted to retain restricted recruitment hours. Where recruitment hours remained within traditional working hours, recruitment remained low with one hospital not receiving any participants.

One hospital offered to participate between the hours of 22:00-07:00 due to competing commercial trial activity associated with an enhanced financial income; commercial trials do not allow co-enrollment so agreement could not be reached, and the site was lost.

### IMP Management

The integration of IMP management, which is bound by the strict oversight of the MHRA, added complexity to existing ambulance service conventional drug management processes. The study protocol, in line with principles of GCP and MHRA legal requirements, defined that accountability logs were to be completed by study-trained paramedics when signing out and signing in IMP on a shift basis, accompanied by regular accountability audits across all participating ambulance services.

Differing medicines management procedures between ambulance services rapidly revealed that a single accountability process as defined by the trial protocol was difficult to generalize across all eight services. Standard medicines management varied between shift-based assignment of drugs bags, assignment of drugs to specific vehicles, and collection and deposit of drug packs at stations or hospitals. The latter, seen in more rural localities, was supported by hospital pharmacies and facilitated exchange at emergency departments.

Three serious breaches relating to IMP were identified and the chief investigator and sponsor self-reported to the MHRA. A serious breach is defined as a noncompliance that is likely to affect to a significant degree the safety or physical or mental integrity of the study participants; and/or the scientific value of the study (34).

The largest breach resulted in a temporary pause to recruitment in one ambulance service following the misplacement of 33 treatment packs, which were unaccounted for during routine accountability checks across stations. Following investigation, the protocol requirement to sign in/out the treatment pack to station each shift misaligned with the trust-specific process where medicines were allocated to, and remained within, specific vehicles. The unfamiliarity with returning trial IMP packs to station drug stores after each shift inadvertently caused IMP packs to be left within vehicles. In view that vehicles would often be moved between stations meant packs would unintentionally be relocated to station drug stores away from their assigned bases. This occurred in the ambulance service participating across its entire geographical area (96 ambulance stations). All packs were traced.

The second and third breaches related to loss of the IMP between ambulance handover in the emergency department and collection by researchers, often when recruiting outside of traditional working hours. RIGHT-2 was unique in comparison with other prehospital trials in that joint participation of ambulance services and hyperacute stroke services was necessary to continue application of IMP for three further days, carry out imaging and in-hospital follow-up. At that time, other prehospital trials have not required the same level of involvement and participation from hospital teams beyond receipt of the patient and data collection (35). Busy emergency departments with high staff rotation, rapid transitioning of patients with some discharged as non-stroke, required early education, frequent communication, and high awareness of the trial, given the relevant infrequency that a patient would be recruited.

Discussions to mitigate recurrence of IMP misplacement within ambulance services included individual issue of IMP to trial paramedics for designated accountability, but strict governance and storage processes meant this was not feasible. Reducing participation to defined stations was considered to increase oversight and control, but recognized this would forfeit recruitment opportunities and the collaboration between hospitals. Finally, assignment of a RIGHT-2 treatment pack to every ambulance for use when required was deemed cost-inefficient as the pool of paramedics participating in RIGHT-2 was low due to its voluntary nature and included the risk of non-trial trained ambulance staff accessing the pack.

To maintain cost efficiencies and ensure pack accountability on station, and recognizing advances in technology, the trial team developed a web-based quick response (QR) code system, with QR codes retrofitted to each treatment pack. The code, when scanned, linked to GPS location determined by the mobile device used. This tracked to the vicinity of the nearest ambulance station as provided by the ambulance service research team. The system gave the user the option to sign in, sign out, allocate a pack to a patient, or notify a pack opened in error or a pack found by non-trial staff with the option to return to the station. Research paramedics within ambulance services had access to this database to track and monitor accountability and compliance, and monitor remote location of packs. Secondarily, this log served as an audit trail but recognizing that during the recruitment phase of 2015-18 not everyone had a device capable of QR code scanning, paper logs were also maintained. This system is now in use with other trials run by the Stroke Trials Unit in Nottingham.

### Data Collection

With consideration given to the relative infrequency of the opportunity to randomize a patient, sample CRFs were available *via* online portals for re-familiarization; nevertheless, many paramedics were completing CRFs for the first time during patient care episodes.

Data were occasionally missed from the CRFs, which required retrospective review of patient care records and backfilling by ambulance service research teams. Omitted data were retrievable with relative ease as these were available through control room logs and patient care records, albeit time consuming for the ambulance service research team to complete.

Reducing the complexity of CRF completion and handover, one ambulance service sought approval for their paramedics to disregard the CRF within the treatment pack. Instead, relevant randomization and consent data were captured on the standard patient care record recognizing most data requested on the CRF were already available elsewhere. These data were extracted by the ambulance service’s research team and submitted on the trial database. Inevitably, this delayed data entry into the trial database and hence its checking and analysis during interim analyses for the purposes of Trial Steering Committee and Data Monitoring Committee review. However, with minor amendments to standard practice this encouraged timelier completion and fewer data corrections for this ambulance service.

At the time of RIGHT-2, a mix of ambulance services used paper and electronic patient data recording. However, all ambulance services are now using electronic patient care records, raising the potential for data to be collected once and shared between the ambulance service and trial database in near real time. This could reduce duplication, transcription errors, and costs. While it is recognized that organizations may use different systems, early engagement will help identify processes to ensure that systems can be put in place and research data can be gathered in a time-efficient way, while providing carefully considered privacy and governance of data. Nevertheless, whether data can be transferred between NHS organizations (i.e., ambulance services and hospital trusts) and outside (e.g., to university trial databases) raises considerable governance issues.

## Discussion

RIGHT-2 was a large, multi-center, ambulance-based randomized controlled trial where paramedics screened and consented 1,149 patients with potential stroke. Treatment was commenced in the ambulance and continued in hospital. As the largest prehospital stroke trial within the UK, multiple challenges emerged throughout the implementation and delivery of RIGHT-2 across eight ambulance services and 54 hospitals. Our findings replicate experiences reported elsewhere (16, 35), but extend these and further explore the intricacies, practical challenges, and steps taken to overcome them.

The setup phase of RIGHT-2 was complex, firstly to coordinate available ambulance service locations with hospital stroke centers willing to participate. Many hospitals had little experience of collaborating in prehospital-initiated research and continuing research that had been commenced within the ambulance-based setting (35). Collaboration with hospital emergency departments and stroke teams proved challenging given the inexperience of many paramedics in research. Initial unease was overcome by early engagement, collaboration, and seeking to simplify processes between key parties. Staggered launches across participating areas sought to deliver assurance before expanding participation.

This approach allowed ambulance services to target specific areas to launch the trial in phases, driving momentum and interest. The frequency of a paramedic attending a stroke is relatively low due to the dispatch model of ambulance services based on priority, timeliness, and distance to call rather than dispatch of a specialist skill set to suspected stroke. Similarly, ambulance dispatchers are not routinely able to assign specific research-trained personnel to specific emergency calls. As UK paramedics voluntarily participate in research, only one-third of paramedics in the participating ambulance services expressed interest in RIGHT-2, as seen previously (36). Further, just 516 of 1487 (35%) of trial-trained paramedics recruited at least one patient. Trial processes needed to be simple, and easy to recall and implement in the often chaotic and time-critical prehospital environment.

The management of medicinal clinical trials commonly presents challenges to trial teams, particularly when seeking practical methods for managing the trial randomization process and associated IMP across multiple settings. Factors specific to the prehospital clinical setting and the treatment being administered may reduce trial protocol compliance and contribute to errors. Time-critical, emergency situations, together with research inexperience, can cause cognitive overload for paramedics who often work alone or in small teams. Workload and time pressures, distractions and interruptions, lack of standardized procedures, and insufficient resources are all major contributors to the risk of medication errors (37). The design of RIGHT-2 attempted to mitigate administration errors as GTN transdermal patches were chosen due to the ease and simplicity of their administration. However, it is inevitable in a time-critical trial with an interface between ambulance stations, ambulance vehicles, and emergency department settings that IMP will be lost, but clearly IMP management in any trial is imperative for consistency and availability.

Our training, retraining, and development of QR code accountability largely removed the problem and provides an easy, efficient system that further trials may be able to benefit from.

Similarly, with recent advances in technology that can support data collection as well as IMP accountability, this may remove some of the inconsistencies and omissions seen in paper-based data recording, as recording trial-based information was a new concept to most paramedics who took part. Three previous prehospital randomized controlled trials in stroke have similarly reported data collection inconsistencies (5, 8, 38). Electronic patient reporting offers the opportunity to harness data collection, prompt for specific data, and potentially extract research-specific detail without adding complexity to the patient care episode, thus reducing mental bandwidth while increasing accuracy of data.

## Conclusion

Ambulance-based research presents several challenges due to the nature and environment of the work and type of incidents attended. This adds complexity to the design and implementation of research in this setting, and early collaboration is essential. The National Ambulance Research Steering Group comprises UK ambulance service research leads and actively encourages early conversations with triallists at the initial stages of proposal development to appraise the viability of ideas and ensure they can be effectively operationalized (https://narsg.uk/)

RIGHT-2 was successful in its primary outcome of testing the feasibility of conducting ambulance-led research, and confirms that early engagement and flexibility in the trial protocol had to be maintained to ensure deliverability and overcome problems at the prehospital and hospital phases.

This work offers real-world insight into the complexity of conducting multi-center, ambulance-based research in a challenging setting; to inform the design and conduct of future ambulance-based trials.

## Data Availability

Data are available upon reasonable request and based on a protocol.

## Trial Registration

Trial registration number ISRCTN26986053.
